# The effect of nanocurcumin on the incidence of atrial fibrillation, and markers of inflammation and oxidative stress level after coronary artery bypass graft surgery: A randomized, double-blind, placebo-controlled clinical study

**DOI:** 10.22038/AJP.2022.20201

**Published:** 2022

**Authors:** Samira Hossaini Alhashemi, Amir Hooshang Mohammadpour, Reza Heidari, Mohammad Hossein Nikoo, Mohammad Hassan Nemati, Afsaneh Vazin

**Affiliations:** 1 *Department of Clinical Pharmacy, School of Pharmacy, Shiraz University of Medical Sciences, Shiraz, Iran*; 2 *Department of Clinical Pharmacy, School of Pharmacy, Mashhad University of Medical Sciences, Mashhad, Iran*; 3 *Pharmaceutical Research Center, Pharmaceutical Technology Institute, Mashhad University of Medical Sciences, Mashhad, Iran*; 4 *Pharmaceutical Sciences Research Center, Shiraz University of Medical Sciences, Shiraz, Iran*; 5 *Department of Cardiology, Medical School, Shiraz University of Medical Sciences, Shiraz, Iran*; 6 *Non-Communicable Diseases Research Center, Shiraz University of Medical Sciences, Shiraz, Iran*; 7 *Department of Cardiac Surgery, Medical School, Shiraz University of Medical Sciences, Shiraz, Iran*

**Keywords:** Cardiovascular diseases, Coronary artery bypass graft Nanocurcumin, Oxidative stress, Postoperative atrial fibrillation

## Abstract

**Objective:**

Postoperative atrial fibrillation (POAF) is the most frequent dysrhythmias observed following coronary artery bypass graft (CABG) surgery. Several studies have shown the beneficial effects of curcumin on cardiovascular diseases; however, there is no clinical trial to examine its effect on POAF. This randomized, double‐blind, placebo‐controlled clinical study was designed to evaluate the prophylactic effects of a nano-formulation of curcumin (SinaCurcumin™) versus placebo on POAF and levels of biomarkers of inflammation and oxidative stress in patients undergoing CABG surgery.

**Materials and Methods:**

A total of 234 eligible patients were randomized to receive 240 mg curcumin nano-formulation or placebo three days prior to the surgery and on the first four postoperative days. The occurrence of POAF was monitored for at least 96 hr after the surgery. Also, C-reactive protein (hs-CRP), malondialdehyde (MDA) and glutathione (GSH) levels were assessed at baseline and the end of the study.

**Results:**

Analyses were done in the intention-to-treat population. No significant difference was observed in the occurrence of POAF between the treatment (9.5%) and placebo (11.5%) groups. Also, curcumin intervention did not alter serum concentration of the hs-CRP, MDA, or GSH in comparison with placebo.

**Conclusion:**

In conclusion, it seems that perioperative treatment with SinaCurcumin™ did not prevent POAF after CABG surgery.

## Introduction

Postoperative atrial fibrillation (POAF) is the most frequent dysrhythmias observed following coronary artery bypass graft (CABG) surgery, with an incidence of approximately 30% (Khan et al., 2019 ▶). POAF may lead to increased morbidity and risk of serious complications including stroke, thromboembolism and multi-organ failure. These events cause prolonged hospital stay and consequently, enhance hospital expenditure (Aranki et al., 1996 ▶; Hravnak et al., 2002 ▶; Nemati and Astaneh, 2016 ▶). POAF occurrence risk factors include advanced age, diabetes mellitus, hypertension, heart failure, cardiac valve disease and left atrial and ventricular hypertrophy (Harada et al., 2015 ▶). Despite several types of research on POAF, the exact mechanism is still unknown. However, several studies have illustrated that inflammation and oxidative stress are the crucial factors in the POAF occurrence (Dobrev et al., 2019 ▶; Nomani et al., 2020 ▶). 

Many studies demonstrated that cardiac surgery activates the inflammation cascade and increase inflammatory markers (Aranki et al., 1996 ▶; Harada et al., 2015 ▶; Dobrev et al., 2019 ▶). Moreover, the results obtained from various investigations have shown a significant association between POAF development and elevated plasma levels of inflammatory biomarkers, including interleukin (IL)-2, IL-6 and C-reactive protein (CRP) (Dobrev et al., 2019 ▶; Nomani et al., 2020 ▶). There is also evidence on the role of pathogenic oxidative stress in the myocardium during CABG surgery (Wu et al., 2015 ▶). Reactive oxygen species (ROS), particularly nicotinamide adenine dinucleotide phosphate oxidase derivatives and monoamine oxidase derivatives, are elevated in the right atria of patients with POAF (Anderson et al., 2014 ▶; Dobrev et al., 2019 ▶; Reilly et al., 2011 ▶). ROS generation leads to atrial remodeling and disruption of electrical activity (Zakkar et al., 2015 ▶). Therefore, anti-inflammatory and antioxidant therapy might be considered a potential preventive treatment for POAF.

Curcumin (diferuloylmethane) is the essential curcuminoid derived from *Curcuma longa, *a popular spice used in Asian foods (Ahangarpour et al., 2019 ▶). Curcumin is considered a safe compound based on several animal trials (Anand et al., 2007 ▶; Naik et al., 2011 ▶) and has demonstrated various therapeutic effects in neurological disorders, cancer, chronic inflammatory diseases and cardiovascular diseases (CVD). Numerous studies illustrated pleiotropic biological activities of curcumin, including anti-inflammatory, anti-oxidant (Vazin et al., 2020 ▶), anti-thrombotic and anti-apoptotic effects (Dastani et al., 2019 ▶). Several studies have demonstrated that curcumin with its anti-inflammatory properties can inhibit the production of tumor necrosis factor- α (TNF-α) and reduce the expression of prostaglandin E_2_, IL-6, and cyclooxygenase-2 genes (Banez et al., 2020 ▶; Boroumand et al., 2018 ▶). These factors have shown a substantial impact on inflammatory processes (Boroumand et al., 2018 ▶). Furthermore, curcumin has been reported to scavenge ROS, inhibit lipid peroxidation and xanthine oxidase and increase the activity of antioxidant enzymes including superoxide dismutase, catalase and glutathione peroxidase (Barangi et al., 2018 ▶; Kukongviriyapan et al., 2016 ▶). Some investigations have shown that curcumin possesses cardioprotective effects and could be effective in improving heart failure, cardiotoxicity, cardiac hypertrophy and diabetic CVD (Li et al., 2020 ▶). In addition, curcumin has demonstrated therapeutic efficacy in hypertension (Hassan et al., 2013 ▶; Li et al., 2019 ▶), hyperlipidemia (Panahi et al., 2018 ▶, 2017) and atherosclerosis (Li et al., 2019 ▶).

The oral absorption and bioavailability of curcumin is low and the compound is rapidly metabolized. The nano-formulation of curcumin with the brand name SinaCurcumin™ has shown significantly higher bioavailability than the conventional powder of curcumin formulations (Rahimi et al., 2015 ▶).

Considering the pathophysiology involved in POAF and the anti-inflammatory and anti-oxidant effects of curcumin, the current double-blind randomized clinical trial aims to evaluate curcumin prophylactic effect of POAF after CABG surgery for the first time.

## Materials and Methods

**Subjects**

This randomized, double-blind, placebo-controlled, multicenter and prospective study was conducted at Ordibehesht and Central Hospitals, both private subspeciality healthcare in Shiraz, Iran, between August 2020 and April 2021. This study was registered at the Iranian Registry of Clinical Trials (registration No. IRCT20200507047327N1) and was approved by an internal review board and the Ethics Committee of Shiraz University of Medical Sciences (IR.SUMS.REC.1399.019).

**Patient selection**

All adult patients undergoing elective on-pump CABG surgery were screened for inclusion criteria. The inclusion criteria were as follows: age between 18 to 82 years old, lack of history of any previous open-heart surgery, taking an angiotensin-converting enzyme inhibitor / angiotensin receptor blocker, statins, aspirin or β-blockers before and after the surgery, and not using antioxidants or anti-inflammatory agents. Patients with cardiac arrhythmias or those taking antiarrhythmic drugs (except β-blockers), or digoxin, having heart valve disorders, heart failure and an ejection fraction ≤ 30%, chronic kidney disease (creatinine ≥ 1.5 mg/dl), severe hepatic insufficiency (increased liver enzymes more than 3 times the upper limit normal), hyperthyroidism, sensitivity to turmeric or individuals who experienced myocardial infarction after surgery were not included. All patients were informed and they signed a written consent. The study was conducted in accordance with the Declaration of Helsinki.

**Allocation, randomization and blinding**

Patients were randomly assigned to two groups using a computer-generated random-digit table (https://www.graphpad.com/quickcalcs). Allocations sequence was implemented by a clinical pharmacist, patients were enrolled by a cardiac surgeon and participants were assigned to either study group by the clinical pharmacist. In this study, patients, the surgeon, nurses and the statistical analyst were blind to allocation of subjects to either group.

**Intervention**

The nano-formulation of curcumin (SinaCurcumin™) was used in this study. SinaCurcumin is a certified curcuminoid product by Exir Nano Sina Company, Tehran, Iran (IRC: 8554358584339287). Soft gelatin capsules containing 80 mg nanocurcumin with the brand name SinaCurcumin™ were prescribed to patients in the intervention group. They received 240 mg nanocurcumin capsules daily (80 mg, three times a day), starting from three days before the surgery and on the first four postoperative days. The individuals in the control group received an equal number of placebo capsules which was prepared by the same company and had a precisely similar appearance as SinaCurcumin™. Both groups had the same surgical premedication and anesthesia protocol and the same cardiothoracic surgeon performed all the on-pump CABG surgeries. Anesthesia was induced by midazolam, sufentanil, and pancuronium bromide and was maintained using sufentanil, midazolam and propofol. 

Baseline information, including demographic characteristics, risk factors, medication history, echocardiographic examination data and laboratory measurements were collected. Additionally, the total length of intensive care unit stay, the drainage volume, the duration of mechanical ventilation and the amount of blood received during the surgery, duration of pump perfusion and duration of the aortic cross-clamp were recorded.

**Primary outcome**

In this study, occurrence of at least one episode of AF persisting more than 5 minutes, was defined as POAF.

. After surgery, the patients were transferred to the intensive coronary care unit where continuous electrocardiographic monitoring was performed for at least 96 hr. The intensive care nurse practitioner reviewed ECG monitoring for the last 24 hr each day. ECG was recorded for assessment by an independent cardiologist blinded to the treatment. 

**Secondary outcome**

Blood samples were collected at baseline and at the end of the study to assess hs-CRP, malondialdehyde (MDA) and glutathione (GSH) levels as biomarkers of inflammation process and oxidative stress, respectively. Serum hs-CRP was assayed using enzyme-linked immunoassay kits (LDN, Nordhorn, Germany). Additionally, serum samples were analyzed for MDA concentrations and total GSH levels (Heidari and Niknahad, 2019 ▶)

**Statistical analysis**

Based on the previous trials, AF during hospitalization has been reported in around 30% of the patients who undergo CABG surgery. At a power of 80% and 5% type I error, 110 patients in each group were needed to differentiate of 15% in the occurrence of POAF between the two study groups. Also, the Kolmogorov–Smirnov test was used in order to ensure the normality of distribution. Analyses were done in the intention-to-treat population, defined as all patients who were randomly assigned to a treatment group. Data are shown as mean values ± standard deviation (SD) for continuous data and percentages for categorical variables. Chi-square test or the Fisher exact test were used to compare categorical variables and the mean variables were compared between the two groups using independent t-test or Mann- Whitney U test. All statistical analyses were performed using the Statistical Package for Social Sciences (SPSS) version 20 (SPSS Inc., Chicago, Ill., USA). A p<0.05 was considered statistically significant.

## Results

**Participants' characteristics**

**Figure 1:**
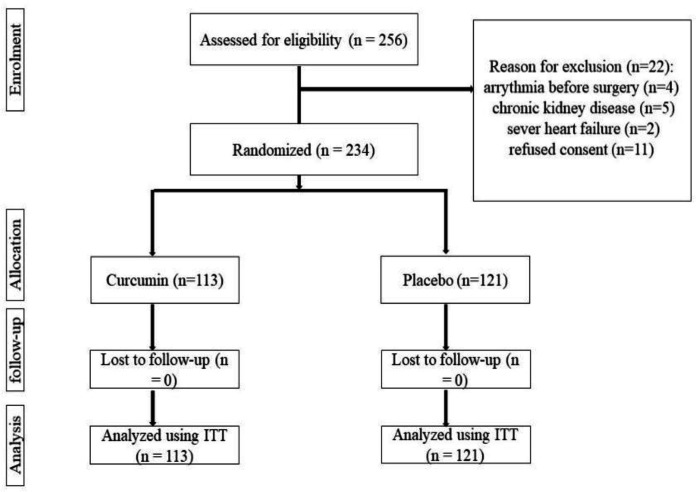
Flow diagram of the study participation. ITT, intention-to-treat

A total of 256 patients who were admitted for CABG surgery at participating hospitals were screened for eligibility. Twenty-two patients did not meet inclusion criteria because of having arrhythmias before surgery (n=4), chronic kidney disease (n=5), and severe heart failure (n=2) or refused to participate in the study (n=11). The remaining 234 patients were randomized to allocate in either group to receive SinaCurcumin™ (n=113) or placebo (n=121). None of the patients were lost to follow-up or were further excluded from the study (Figure 1). The 234 patients who were randomly assigned constituted the intention-to-treat population.

The demographic and clinical characteristics of the two groups are depicted in Table 1. There were no significant differences in age, sex, body mass index (BMI), smoking, serum creatinine levels, left ventricular ejection fraction (LVEF), concomitant diseases (e.g. diabetes mellitus, hyperlipidemia or hypertension), or resumption of postoperative medications (p>0.05). The procedural variables were also compared between the two groups and are reported in Table 2. The differences between the study groups concerning cross-clamp, pump duration, the drainage volume in the intensive care unit, intubation time and length of hospital stays showed no statistically significant differences (p>0.05). 

**Table 1:** Baseline clinical and demographic characteristics

	**nanocurcumin (n=113)**	**Placebo (n=121)**	**p**
Age, years, mean (SD)	61.56 (8.35)	62.35 (9.2)	0.49^a^
Male sex, n (%)	80 (70.8 %)	78 (64.5%)	0.30^b^
BMI, Kg/m^2^, mean (SD)	27.15 (4.5)	26.75 (5.4)	0.54^a^
Creatinine, mg/dl, mean (SD)	0.9 (0.4)	1 (0.5)	0.09^a^
LVEF, percentage, (SD)	45.5 (8.4)	46.8 (9.1)	0.26^a^
Smoking (%)	35.1	32.4	0.66^b^
Diabetes mellitus (%)	45.4	49.3	0.55^b^
Hypertension (%)	62.7	65.3	0.68^b^
Hypercholesterolemia (%)	66.2	54.2	0.06^b^
Postoperative beta blocker (%)	100	100	1.00^b^
Postoperative statin (%)	100	100	1.00^b^
Postoperative ACE inhibitors/ARB (%)	85	80	0.32^b^

BMI, body mass index; LVEF, left ventricular ejection fraction; ACE angiotensin-converting enzyme; ARB, angiotensin receptor blocker.

**Main outcome**

Postoperative AF was developed in twenty-five patients (11%). The incidence of POAF was 9.5% and 11.5% in SinaCurcumin™ and placebo groups, respectively. Although the occurrence was lower in the treatment group, no significant differences between the groups was observed in this regard (p=0.62) (Table 2). Two of the patients had POAF on the day of surgery and the rest of the POAFs occurred on the 2^nd^ and 3^rd^ day after the surgery. Table 2 also demonstrates the occurrence of premature ventricular complexes, short-term ventricular tachycardia, and premature ventricular complexes. The results demonstrated that there was no significant difference between the placebo and SinaCurcumin™ groups (p>0.05).

**Table 2:** Comparison of the patients’ procedural characteristics and clinical outcomes

	**Nanocurcumin (n=113)**	**Placebo (n=121)**	**p**
**Aortic cross-clamp, min, mean (SD)**	32.42 (7.92)	33.57 (6.49)	0.22^a^
**Pump duration, min, mean (SD)**	51.58 (9.4)	53.37 (9.7)	0.09^a^
**Drainage in ICU, ml, mean (SD)**	780.25 (332.97)	748.57 (276.58)	0.43^a^
**Intubation time, h, mean (SD)**	14.4 (2.27)	14.99 (1.87)	0.91^a^
**Inotrope usage, n (%)**	5 (4.5%)	6 (4.95%)	0.87^b^
**ICU blood transfusion, n (%)**	49 (43.5%)	62 (51%)	0.25^b^
**Length of hospital stays, days, mean (SD)**	5.18 (0.65)	5.32 (0.57)	0.08^a^
**Occurrence of AF, n (%) ** **(N=25, 11%)**	11 (9.5%)	14 (11.5%)	0.62^b^
**Premature ventricular complexes (%)**	100%	100%	1.00^b^
**Short-term ventricular tachycardia (%) **	23%	18%	0.34^b^
**Premature atrial complex (%)**	100%	97.5%	0.09^b^

ICU, intensive care unit; AF, atrial fibrillation. ^a^ T-test was used. p<0.05 was considered statistically significant. ^b^ Chi-square test was used. p<0.05 was considered statistically significant.

**Table 3:** hs-CRP, MDA and GSH levels at pre-and post-operation times in both groups

		**nanocurcumin (n=113)**	**Placebo (n=121)**	**p**
**hs-CRP, mg/L, mean (SD)**	Baseline	0.6 (0.2)	0.6 (0.15)	1.00
	Post-CABG	13.2 (5.3)	13.9 (6.3)	0.36
	change	12.56 (5.5)	13.32 (6.7)	0.35
**MDA, µmol/L, mean (SD)**	Baseline	7.31 (1.4)	7.27 (1.5)	0.83
	Post-CABG	8.6 (1.5)	9 (1.8)	0.07
	change	1.35 (1.5)	1.6 (1.4)	0.19
**GSH, µmol/L, mean (SD)**	Baseline	450.4 (98.6)	440.5 (111.9)	0.47
	Post-CABG	378.4 (80.6)	362.9 (81.3)	0.14
	change	-75 (60.2)	-77.8 (75)	0.75

Hs-CRP, high sensitivity C-reactive protein; MDA, malondialdehyde; GSH, glutathione.

T-test was used. p<0.05 was considered statistically significant.

**Inflammation marker**

As shown in Table 3, no significant difference between the control and SinaCurcumin™ groups was observed in baseline level of hs-CRP (p=1.00). Seven days of treatment with nanocurcumin did not alter serum concentration of hs-CRP after the surgery (p=0.35). 

**Oxidative stress markers**

The postoperative serum GSH levels in nanocurcumin group did not show a significant difference compared with the placebo group (p=0.14). Also, supplementation with 240 mg nanocurcumin did not lead to any significant difference in serum MDA levels (p=0.07) (Table 3)

## Discussion

In the present investigation, the effect of oral administration of nanocurcumin (SinaCurcumin™) on prevention of AF after CABG surgery, was evaluated. Administration of 240 mg SinaCurcumin™ capsules daily, pre- and post-operative, failed to prevent arrhythmias and length of hospital stay. Biomarkers of inflammation and oxidative stress showed no significant difference between the placebo and SinaCurcumin™ groups.

POAF as the most frequent dysrhythmias observed after CABG surgery, may lead to increased morbidity, mortality and cost of postoperative care. Thus, it is important to prevent post-CABG AF. Previous researches revealed the effect of curcumin on CVDs. Pre-treatment with curcumin nanoparticles (100, 150 and 200 mg/kg BW) given for 15 days, has been reported to prevent QT prolongation and QRS complex enlargement as well as cardiomyocyte damage in isoproterenol-induced myocardial infarction in rats (Boarescu et al., 2019 ▶). In addition, curcumin is a multi-ion channel blocker decreased the incidence and average duration of ventricular arrhythmias resulted from ischemia–reperfusion injury in rabbit ventricular myocytes (Barangi et al., 2018 ▶; Song et al., 2020 ▶). Brosková et al. discovered that curcumin alleviates reperfusion-induced dysrhythmias in a rat model of the heart and mesenteric ischemia-reperfusion (Brosková et al., 2013 ▶). Curcumin can improve ventricular tachyarrhythmias induced by Brugada syndrome (Keller et al., 2005 ▶). On the other hand, Dastani et al. indicated that nanocurcumin given at the dose of 80 mg/day for 5 days, did not affect the left ventricular function or incidence of various arrhythmias in the patients with unstable angina (Dastani et al., 2019 ▶). 

Several studies have found a significant association between the inflammatory response and incidence of POAF (Dobrev et al., 2019 ▶). Inflammatory response mediators can induce structural and electrical remodeling leading to AF (Nomani et al., 2020 ▶). Most studies have shown the association between pre- and postoperative CRP levels with the POAF occurrence (Dobrev et al., 2019 ▶; Khan et al., 2019 ▶). Some investigations indicate that curcuminoids have anti-inflammatory and anti-oxidative effects on CVDs (Li et al., 2019 ▶; Li et al., 2020 ▶). Previous studies have illustrated that 1500 to 3000 mg of conventional curcumin has an anti-inflammatory effect (Adibian et al., 2019 ▶; Rahiminia et al.,2015 ▶); moreover, Hatamipour et al. compared curcumin nano-formulation with conventional form and found that 80 mg of nanocurcumin was equivalent to 1 g of conventional curcumin (Hatamipour et al., 2019 ▶). Therefore, in the current study, 240 mg per day of nanocurcumin, which is equivalent to 3 g of conventional curcumin, was used. However, there are controversial observations from the studies conducted in the different populations and applying different doses. For example, 80 mg/day nanocurcumin supplementation in overweight non-alcoholic fatty liver disease patients for 3 months improved glucose indices and lipids and decreased TNF-α, hs-CRP, and IL-6 (Jazayeri-Tehrani et al., 2019 ▶). However, there are some controversial findings on the effectiveness of curcumin in such conditions. For instance, a study was performed in patients with coronary artery disease to investigate the efficacy of curcumin on some cardiovascular risk factors. It was found that 2 g/day curcumin for 8 weeks could attenuate the concentration of serum triglyceride, LDL-cholesterol and VLDL-cholesterol, however, the level of serum hs-CRP remained unchanged (Mirzabeigi et al., 2015 ▶). A same result was also documented in another clinical study after supplementation with 1 g curcumin for 4 weeks. Besides, our result is consistent with the result of Karimi et al. (2020) ▶ in which, 160 mg of nanocurcumin supplementation for 10 days did not show any considerable effect on hs-CRP in the serum of patients with sepsis. Effect of curcumin on nuclear factor kappa-light-chain-enhancer of activated B cells (NF-κB) could be the probable reason for such results. This protein complex is a critical transcription factor modulating cytokines and proteins gene expression following exposure to inflammatory stimuli. Curcumin has been shown to have an anti-inflammatory effect by down‐regulation of NF-κB activation, other intracellular signaling proteins and cytokines gene expression (Banez et al., 2020 ▶). It seems that the short duration of treatment of the present trial could be considered one of the probable reasons for observing no significant changes in measured factors between the two groups. 

Several studies have investigated the impact of ROS generation in developing POAF (Zakkar et al., 2015 ▶). The effect of curcumin on oxidative stress may occur through ROS-scavenging activity of its phenolic group and its potential to increase antioxidant enzymes (Banez et al., 2020 ▶). For example, clinical research in type 2 diabetic patients suggested that curcumin supplementation for 8 weeks could enhance the total antioxidant capacity and decrease the level of MDA (Panahi et al., 2017 ▶). In another study, consumption of nanocurcumin in forty-four women with metabolic syndrome for 6 weeks, reduced the inflammation biomarkers and MDA concentration (Osali, 2020 ▶). However, 14 days of curcumin administration before the cardiac surgery could not cause significant differences in MDA or GSH concentration between the groups which is consistent with results obtained in the present study (Sukardi et al., 2016 ▶).

Another explanation for having no significant difference between the nanocurcumin and placebo groups is that the POAF happened in 25 cases (11%), including 9.5 and 11.5% of patients in the nanocurcumin and placebo groups, respectively. The low occurrence of AF after surgery is not only associated with development in the surgical techniques but it is also associated with some other variables such as the exclusion of some patients including individuals with heart valve disorders, heart failure, or chronic kidney disease and those who needed emergency surgery, from the study.

Also, there are some limitations in the present study restricting the results. First, the short duration of the treatment and the early termination of the trial due to the designed primary endpoint. Another limitation of the study was that cytokines and transcription factors gene expression was not evaluated. According to previous studies, these factors have shown an impact on the incidence of POAF. Also, the two groups in this study were given propofol by continuous infusion for maintenance of anesthesia. Since propofol has shown anti-arrhythmic properties, the results may be affected by this medication. Since there was no clinical trial to evaluate prophylactic effects of nanocurcumin on POAF, the sample size was determined based on the incidence of AF in previous studies, while occurrence of POAF in the current study was 11%. Thus, sample size in future study should consider this finding.

Based on previous studies on the molecular mechanism of POAF, the role of inflammation in this pathological condition and anti-inflammatory properties of curcumin, it seems that the drug regimen used in this study was not sufficient to induce anti-inflammatory reactions. The short-time schedule between the beginning of therapeutic intervention and surgery might have been insufficient. Therefore, this research may open a new avenue for the researchers to focus on finding the appropriate time period to be considered between starting the drug and the surgery. Therefore, other research groups can find the profile of anti-inflammatory properties of the drug versus time and then it could be used as a guide for the clinicians to use such therapeutics.

It seems that perioperative treatment with 240 mg SinaCurcumin™ (80 mg, three times per day) did not prevent POAF after CABG surgery. The current research is the first report on the efficacy of curcumin against POAF. Although this study has improved our understanding about curcumin effects on cardiovascular surgery, more human studies are needed to confirm curcumin’s influence on POAF and determine an effective dose and treatment duration.

## Conflicts of interest

The authors have declared that there is no conflict of interest.
